# Quality of Life Theory III. Maslow Revisited

**DOI:** 10.1100/tsw.2003.84

**Published:** 2003-10-13

**Authors:** Soren Ventegodt, Joav Merrick, Niels Jorgen Andersen

**Affiliations:** The Quality of Life Research Center, Copenhagen K, Denmark

**Keywords:** Quality of Life, QOL, Maslow, life purpose, life mission theory, self-actualization, human development, holistic medicine, public health, Denmark, etiology

## Abstract

In 1962, Abraham Maslow published his book *Towards a Psychology of Being*, and established a theory of quality of life, which still is considered a consistent theory of quality of life. Maslow based his theory for development towards happiness and true being on the concept of human needs. He described his approach as an existentialistic psychology of self-actualization, based on personal growth.When we take more responsibility for our own life, we take more of the good qualities that we have into use, and we become more free, powerful, happy, and healthy. It seems that Maslow's concept of self-actualization can play an important role in modern medicine. As most chronic diseases often do not disappear in spite of the best biomedical treatments, it might be that the real change our patients have for betterment is understanding and living the noble path of personal development. The hidden potential for improving life really lies in helping the patient to acknowledge that his or her lust for life, his or her needs, and his or her wish to contribute, is really deep down in human existence one and the same. But you will only find this hidden meaning of life if you scrutinize your own life and existence closely enough, to come to know your innermost self.

## INTRODUCTION

In 1962, when Abraham Maslow published his book *Towards a Psychology of Being*[1], there were hardly many who could have guessed that by doing this he had established a theory of quality of life, which still even after 40 years is considered a consistent theory of quality of life. Maslow based his theory for development towards happiness and true being on the concept of human needs. He described his approach as an existentialistic psychology of self-actualization, based on personal growth.

As an American psychologist, Abraham Maslow characterized the good life as a fulfillment of needs, which is one of eight different ways of considering quality of life that have been eagerly used throughout history[2]. His perspective was simple: happiness, health, and ability to function come when you take the responsibility for fulfilling all your needs. The difficulty in this lies in the fact that to do so you must know yourself well enough to understand which needs you really have.

Maslow tried to solve this difficult problem by giving a universal roadmap of personal development, applying a progressive series of needs, where the next need is revealed as you realize the previous. In this way, Maslow established a form of staircase, which obtained its popular interpretation in the pyramid or his hierarchy of needs (see Fig. 1).

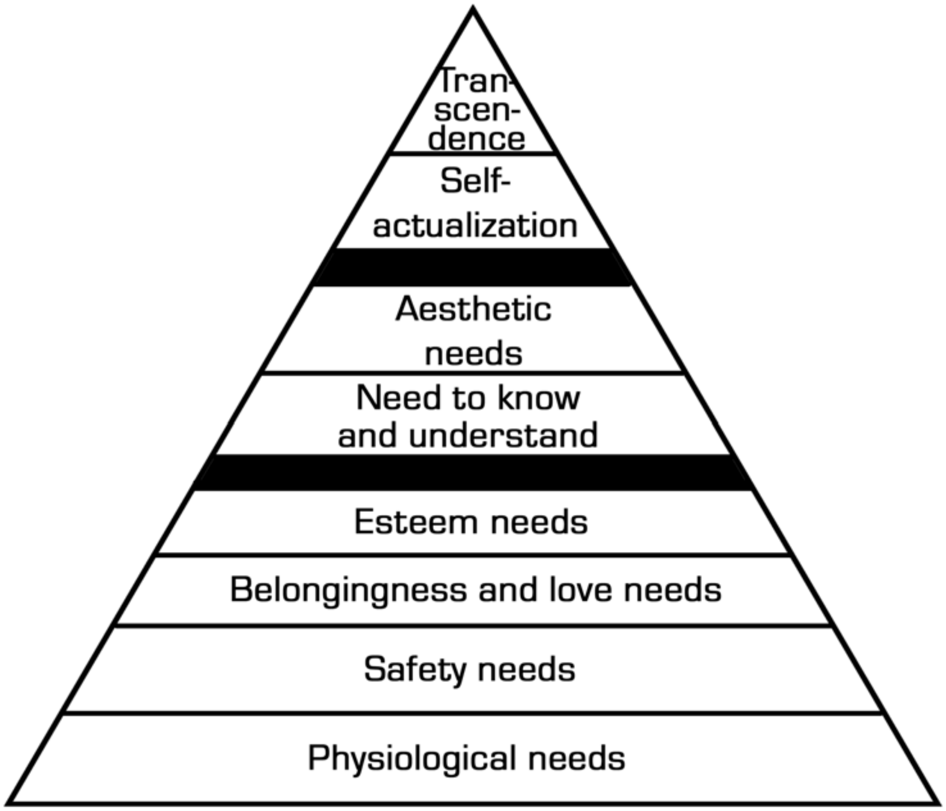

**FIGURE 1**. Maslow's hierarchy of needs.

Maslow described the ideal life as a long journey through the eight needs, which takes its departure from the concrete and down to earth to the abstract and divine — transcendent in his own word. In order to fulfill them one by one, we must develop our beings to be more spontaneous, independent, active, and responsible. Seen in the light of the life mission theory, which claims that every man has a huge and fundamental talent that can be realized both in private and professional life, is it possible that Maslow's theory can be given a new and simple interpretation.

## THE HIERACHY OF NEEDS

In the bottom of the hierarchy we find the four most basic needs of the human being:

- The physiological needs, like food, clothes, and sleep
- The need for peace of mind, like a safe residence
- The need for love as, for instance, to belong to someone
- The need for respect or to be acknowledged

In the middle of the hierarchy we find two more advanced needs:

- The need for knowledge and understanding — to know ourselves and to understand our world
- The need for creativity and aesthetics — to use our knowledge and talents to create

In the top of the hierarchy we find our two most abstract needs:

- The need for self-actualization — to realize our personal meaning of life
- The need for transcendence — to become an integrated and valuable part of the world

## A PATH TO HAPPINESS

Maslow believed that the truly happy person would have these eight needs fulfilled. Unfortunately he himself only knew a handful who had succeeded. So self-actualization and transcendence was in Maslow's understanding a rarely obtained, almost utopian, state of being.

In spite of all the possibilities and the great freedom of modern man, it looks like most of the people in our society today still do not surpass level 3 or 4 in the hierarchy. Most people seem to live their lives in search for love and respect, mostly without getting it. Maybe the time has now come, bearing in mind the increasing economic wealth in the western part of the world, where we can have all our needs fulfilled, from the most concrete to the most abstract.

When we understand what our needs are and that it is our own responsibility to fulfill them, a miracle can happen: we can transform from bitter and troubled human beings to happy, easygoing people with smooth characters, being great friends and the heroes of our children. When we take more responsibility for our own life, we take more of the good qualities that we have into use, and we become more free, powerful, happy, and healthy.

So how is this done? Maslow provides us with a masterly plan.

## LOVE AND CARE

Love is our basic relationship to the other, to thou, so well described by Martin Buber in *I and Thou*[3]. When we have problems in receiving love from others, the explanation is quite simple according to most existential philosophers: we do not love ourselves sufficiently, and it then becomes almost impossible to really love someone else or to receive love from others. This is because it unconsciously reminds us of painful days in our own past, when we were not given the love we needed. So we are all wounded children. But there is a solution to this problem.

As caring physicians, we often try to help our patients to get relief from the severe existential problems such as the feeling of loneliness so common in the western world that follows a "closed heart". If we are willing to give full-hearted holding to such a patient, he or she often spontaneously reveals a number of very painful, almost unbearable, events in life where love, care, and concern came in short.

Children often react to an overwhelming, emotional pain by making negative decisions, which serve the purpose of adapting by changing the focus and whole perspective of life, thereby liberating the child from the suffering. If parents do not show their love and affection for their youngsters for a period of time, the children often conclude that it is because they are not worthy of love and thinking that, it becomes understandable why and also tolerable that, nobody loves them.

Most people have that kind of life-rejecting decision buried in their subconsciousness. As we grow older, these decisions accumulate to undermine our self-esteem and self-confidence. Maybe we were criticized in school and concluded that we were not intelligent. Maybe we received less love than our younger brother and sisters, and concluded that we were less worthy as human beings, or even bad persons. As we experience our difficulties in life, we generally adapt by making one negative decision after another.

Therefore, our worldview and self-perception often grows negative little by little. It is not a coincidence that many people end up conceiving that they and their surrounding world are less than optimal. It is a simple consequence of the normal conclusions from the unpleasant experiences of our childhood. As shown in several population studies[4,5] and explained by the life mission theory[6], such negative attitudes are very common, present with 25% of the population at least, and strongly correlated with poor physical and mental health.

In order to heal the wounds of our soul and to get rid of past sufferings, we have to go back and finish off the troublesome episodes, with a new understanding of life. Therapy, which gives us the love that we did not receive earlier, can help us to give love, just as we can deal with the problem ourselves if we have the resources, for instance in writing our biography in every detail. Love is a basic human talent that cannot really be lost, just forgotten. With the right motivation, everyone can learn to love again. The barrier is that you must go back and feel the original pain, understanding what it is about and, finally, you must let go of the accumulated negative decisions.

## RESPECT

Respect comes from acknowledging that you know that I am I, you are you, and the other is the other. You own your problems, as I own mine. You are fully competent to solve your problems and I can solve mine. Everyone is intelligent and has his own resources to find his own solutions to his problems.

Respect is often the main topic in much psychotherapy[7], because respect often is in short supply from birth and on. Sometimes parents in our society believe that babies are not intelligent just because they cannot speak, but intelligence and meaning is not based on words. How could we learn to speak in the first place if we could not understand without the use of words?

In meeting other people with respect, we help them to see, acknowledge, and use their own resources: intelligence, tacit knowledge, joy of life, personal energy, and direction. It is only when we respect ourselves that we can access the hidden resources of our life. This is why self-respect is such an important precondition in order to have our next need fulfilled, which is knowledge and understanding.

## KNOWLEDGE

Many people have experienced the sensation of knowing something, as sweet as candy. In science we talk about “sweet science”, described in an intriguing way by the Nobel Prize winner Francis Crick[8] in relation to the discovery of the DNA double helixes. “Sweet science” is the term used for the peculiar and profound happiness and satisfaction obtained in exploring and getting scientific understanding, and philosophy, for instance, is translated from Greek to "love to wisdom".

We believe that everyone can experience the sweetness and happiness of gaining wisdom when we find the deep and genuine need inside us that is all about being conscious about life, ourselves, and the world we live in. Actually being, knowledge, and happiness often go hand in hand. In ancient India people talked about reaching the level of existence called "sat-sit-ananda": beingness, wisdom and happiness as one. When we know the happiness that comes with knowing and understanding, we reach a new and deeper need sometimes called creativity.

## CREATIVITY

Without a deep and coherent knowledge about the world and yourself, creativity becomes flat like a penny. But standing on the shoulders of a real, true, and profound knowledge is a natural and spontaneous urge to be creative. All the knowledge and understanding must be taken into use, to create something new. The experience of true creativity has been described in a intriguing way by Csikszentmihalyi as being in a "flow"[9].

When we create, we use our talents. Talent means having a potential for being creative. We have a natural predilection for using our knowledge and ourselves to create value in the context where we find ourselves. Some have a talent for practical things, others for art and aesthetics. Others again have talents for helping others or for bringing truths to the world. All humans have huge talents that are often hidden, even though we are born to create and contribute in order to make this world a better place.

There are a lot of ill-fated artists and scientists and there are a lot of creative human beings that end up breaking down. That is because knowing and creating is really not enough to make you a happy person. You have to find your calling, your personal mission of life, your ultimate goal, or should we say… the meaning of your life.

## THE LIFE MISSION

Our seventh need is the need of self-actualization. Many people seem to have misinterpreted this. It is about becoming real and present here and now, and has nothing to do with becoming a self-satisfied, ruthless egoist. There is a profound and almost esoteric meaning in this need: everyone has got something that potentially can make him or her happy. This need is the meaning of life itself. In the life mission theory it is called the purpose of life, or the personal mission[6]. This mission is an individual thing as everyone has his or her own mission; its final formulation is often first found after years of exploring the debts of the soul.

When we have found our personal mission and put it into words, life can become much easier. Our whole perspective of life can now change. The problems in our work-life and relationships can suddenly be seen in a new light: "So this is why!" With this new ability to understand the inner logic of one's own life, it is quite easy to start living with focus on what is beautiful, good, and true. Suddenly we stop serving outer purposes and we start serving our inner truth. Ultimately we will be the balanced and independent people that can have mature and fruitful relations with the outside world.

We have now found the course of our life. When patients spontaneously get healed from what seems to be incurable cancer, research seems to indicate that the spontaneous remission is often a fruit of such a complete and radical change of course of life towards meaning and inner truth[10,11,12].

## TRANSCENDENCE OR COMING HOME IN THE UNIVERSE

Transcendence is one of the difficult words in our language. It means, "crossing the limits", going beyond our boundaries. In Maslow's interpretation of the word, transcendence means being present in such a way that we forget ourselves and melt away into the world that surrounds us. Living our lives in coherence with our nature and primary talent makes us champions in our own league. This championship always seems to have one purpose: that we contribute to the world. When we do this flawlessly, we have reached what seems to be our natural state of being. We have come home in existence and home in the world. Transcendence is the inevitable sign that self-actualization is coming well about.

## DISCUSSION

Maslow's hierarchy of needs has met severe criticism from researchers arguing that it is not in accordance with facts. We are often fighting and keeping our direction in spite of not getting our needs fulfilled. Just think of soldiers, creative in spite of lack of security, financially pure artists, and socially isolated scientists. Hungry children are still playing, so needs cannot be ordered in such a hierarchy, argues the Danish psychiatrist, Anton Aggernæs[13].

In a way, this criticism is taken care of by the life mission theory[6], so that both Maslow and Aggernæs are right. Deep down we are always striving to actualize ourselves no matter what, but during hard times we have to modify ourselves, often to such a degree that we almost completely loose contact with our own purpose of life. This adaptation through modification of our personality and worldview seems highly advantageous for our early survival and every child seems to use it eagerly.

Later in life, when our life conditions become better, we can wake up and heal our imbalances and spiritual wounds, and get rid of our impurities and lies. This is what Maslow called personal growth, and it might be exactly what our patients need in order to become well again.

Biomedicine helps the patient by supporting body and mind, where the symptoms and weaknesses appear. Maslow's philosophy concerning consciousness, existentialistic choices, personal growth, and self-actualization points to another way of helping our patient. We can help him or her to improve quality of life, health, and general ability to work and function. We can do this by supporting the wish of our patients to assume responsibility for his or her existence and own needs. The questions we must ask to help our patient develop his or her consciousness and take more responsibility are, regardless of age, quite simple: "What do you need?" and "How will you get it?"

But there is more to be done than just asking wise questions; the patient often needs a period of intense holding. As physicians we have to meet our patients historically frustrated needs with love, care, respect, and acknowledgment of their unique personality, talent, and nature. Without holding, the patient might not dare to start the exciting, but often difficult, voyage it is to deal with the force of old habits and views, take responsibility for their own troubled lives, and/or start the process of self-actualization.

If it is a growing child or a youngster, the care received from a physician could be the key factor that this child needs to overcome a crisis and thus prevent the child from stagnating in his or her development.

A vigorous development and the unfolding of life often lead to a better health. Maybe this improvement of one's quality of life by assuming more responsibility is the best medicine in the world. The existing research in this field is at present scarce, but seems promising. It looks as though personal development and increased responsibility for one's quality of life seems to be the main factor in remarkable psychosomatic studies[11,12]. A pilot study in quality of life as medicine, where a group of patients with incurable chronic pains was encouraged to assume more responsibility for their own life and their fulfillment of needs, showed that the patients could let go of half of their pains and other inconveniences in just a few months [14]. Maybe *quality of life*

*as medicine* ought to be a theme for thorough scientific research, and maybe this research should be given high priority as personal development and self-actualization is cheap for society, and indeed beneficial for the patient.

## CONCLUSION

It seems that Maslow's concept of self-actualization can play an important role in modern medicine. As most chronic diseases often do not disappear in spite of the best biomedical treatments, it might be that the real change our patients have for betterment is understanding and living the noble path of personal development, rediscovered and explained by Abraham Maslow half a century ago.

Maslow's hierarchy of needs makes the process of empowering our patients more understandable, simple, and easy to take into clinical use. It seems to be a useful tool for the physician, who wishes to help the patient mobilize his or her hidden potentials. As Maslow's hierarchy can be explained in a simple and straightforward manner to any patient, he or she can get a route to improve quality of live. The patient can incorporate it in his general plan of life, and eventually obtain happiness and healing.

The hidden potential for improving life really lies in helping the patient to acknowledge that his or her lust for life, his or her needs, and his or her wish to contribute is really deep down in human existence one and the same. But you will only find this hidden meaning of life if you scrutinize your life and existence closely enough, to come to know your innermost self.

## ACKNOWLEDGMENTS

This study was supported by grants from The 1991 Pharmacy Foundation, as well as by supplementary grants from Goodwill-fonden, the JL-Foundation, E. Danielsen and Wife's Foundation, Emmerick Meyer's Trust, the Frimodt-Heineken Foundation, the Hede Nielsen Family Foundation, Petrus Andersens Fond, Wholesaler C.P. Frederiksens Study Trust, Else & Mogens Wedell-Wedellsborg's Foundation, and IMK Almene Fond. We gratefully acknowledge the critical scrutiny and expert linguistic assistance of Ib Ravn, Ph.D. The research was approved by the Copenhagen Scientific Ethical Committee under number (KF)V.100.2123/91.

## BIOSKETCHES

**Søren Ventegodt, MD,** is the Director of the Quality of Life Research Center in Copenhagen, Denmark. He is also responsible for a Research Clinic for Holistic Medicine in Copenhagen and is a popular speaker throughout Scandinavia. He has published numerous scientific or popular articles and a number of books on holistic medicine, quality of life, and quality of working life. His most important scientific contributions are the comprehensive SEQOL questionnaire, the very short QoL5 questionnaire, the integrated QOL theory, the holistic process theory, the life mission theory, and the Danish Quality of Life Research Survey, 1991–94 in cooperation with the University Hospital of Copenhagen and the late pediatric professor Bengt Zachau-Christiansen. E-mail: ventegodt@livskvalitet.org. Website: www.livskvalitet.org/

**Joav Merrick, MD, DMSc,** is Professor of Child Health and Human Development affiliated with the Zusman Child Development Center and Division of Community Health at the Ben Gurion University, Beer-Sheva, Israel and presently the Medical Director of the Division for Mental Retardation, Ministry of Social Affairs, Jerusalem and the Director of the National Institute of Child Health and Human Development. He has numerous publications in the field of child and human development, rehabilitation, intellectual disability, disability, health, welfare, abuse, advocacy and prevention. Dr. Merrick received the Peter Sabroe Child Award for outstanding work on behalf of Danish Children in 1985 and the International LEGO-Prize ("The Children's Nobel Prize") for an extraordinary contribution towards improvement in child welfare and well being in 1987. E-mail: jmerrick@internet-zahav.net. Website: www.nichd-israel.com

**Niels Jørgen Andersen, MSc,** Professor, Department of Innovation and Economic Organization, Norwegian School of Management. This department conducts research and provides teaching in central topics related to innovation, business development, management of global companies, business history, and economic organization. Research activities within the Department are related to four core subjects within the discipline: business history, cooperative organizations, business development and entrepreneurship, and finally studies of industries with a special focus on the electricity industry. He is also the dynamic chairman of the nonprofit organization Stiftelsen Holistisk Medisin Scandinavia, which aims to support the scientific development, research, and documentation of complementary and holistic medicine in Scandinavia. E-mail: niels.j.andersen@bi.no. Website: www.bi.no/users/fgl93013/
